# Immediate Effects of tDCS on the μ-Opioid System of a Chronic Pain Patient

**DOI:** 10.3389/fpsyt.2012.00093

**Published:** 2012-11-02

**Authors:** Marcos Fabio DosSantos, Tiffany M. Love, Ilkka Kristian Martikainen, Thiago Dias Nascimento, Felipe Fregni, Chelsea Cummiford, Misty Dawn Deboer, Jon-Kar Zubieta, Alexandre F. M. DaSilva

**Affiliations:** ^1^Headache and Orofacial Pain Effort, Biologic and Materials Sciences Department and MCOHR, School of Dentistry, University of MichiganAnn Arbor, MI, USA; ^2^Faculdade de Medicina, Universidade Federal do Rio de JaneiroRio de Janeiro, Brazil; ^3^Translational Neuroimaging Laboratory, Molecular and Behavioral Neuroscience Institute, University of MichiganAnn Arbor, MI, USA; ^4^Laboratory of Neuromodulation, Spaulding Rehabilitation Hospital, Harvard UniversityBoston, MA, USA

**Keywords:** tDCS, PET, opioid receptors, neuroplasticity, trigeminal neuropathic pain, post-herpetic neuralgia

## Abstract

We developed a unique protocol where transcranial direct current stimulation (tDCS) of the motor cortex is performed during positron emission tomography (PET) scan using a μ-opioid receptor (μOR) selective radiotracer, [^11^C]carfentanil. This is one of the most important central neuromechanisms associated with pain perception and regulation. We measured μOR non-displaceable binding potential (μOR BP_ND_) in a trigeminal neuropathic pain patient (TNP) without creating artifacts, or posing risks to the patient (e.g., monitoring of resistance). The active session directly improved in 36.2% the threshold for experimental cold pain in the trigeminal allodynic area, mandibular branch, but not the TNP patient’s clinical pain. Interestingly, the single active tDCS application considerably decreased μORBP_ND_ levels in (sub)cortical pain-matrix structures compared to sham tDCS, especially in the posterior thalamus. Suggesting that the μ-opioidergic effects of a single tDCS session are subclinical at immediate level, and repetitive sessions are necessary to revert ingrained neuroplastic changes related to the chronic pain. To our knowledge, we provide data for the first time *in vivo* that there is possibly an instant increase of endogenous μ-opioid release during acute motor cortex neuromodulation with tDCS.

## Background

Pain is described as a complex experience affecting not only the sensory, but also the affective and cognitive systems (Merskey and Bogduk, 1994). Although the central mechanisms involved in pain perception and modulation have not been completely elucidated, recent years have seen significant advances in the understanding of the anti-nociceptive mechanisms controlling the pain experience in humans. One of the most important modulatory mechanisms is the endogenous opioidergic system, which is involved in the regulation of experimental and clinical pain, as well as in the effects of analgesic opiate drugs. Studies with positron emission tomography (PET) have shown decreased opioid receptor non-displaceable binding potential (BP_ND_) in patients with chronic pain disorders, including rheumatoid arthritis (Jones et al., 1994), neuropathic pain (Maarrawi et al., 2007a; DosSantos et al., 2012), and fibromyalgia (Harris et al., 2007) when examined with both selective for μ-opioid receptor (μOR; Harris et al., 2007; DosSantos et al., 2012) and non-selective (Jones et al., 1994; Maarrawi et al., 2007a) opioid receptor markers. The data available points to either or both endogenous opioid release, and down-regulation of opioid receptors. It has also been demonstrated that sustained pain activates μOR mediated neurotransmission in a complex network of brain areas related to pain, including the dorsolateral prefrontal cortex, anterior cingulate, anterior and posterior insula, thalamus, hypothalamus, amygdala, and periaqueductal gray matter. Furthermore, the magnitude of these regional activations was related to the individual’s capacity to suppress sensory and affective elements of the pain experience (Zubieta et al., 2001).

Therapies that directly modulate brain activity in specific neural networks might be particularly suited to relieve chronic pain. Interestingly, a novel method of non-invasive brain stimulation, namely transcranial direct current stimulation (tDCS), has been reported to produce lasting therapeutic effects, when applied to the motor cortex, in chronic pain disorders, including fibromyalgia (Fregni et al., 2006; Riberto et al., 2011), orofacial pain attributed to viral infection (Antal and Paulus, 2011), and chronic migraine (DaSilva et al., 2012). This technique is based on the application of a weak direct current to the scalp that flows between two electrodes (anode and cathode). Some studies have shown that the efficacy of tDCS depends critically on parameters such as electrode position and current strength (Nitsche et al., 2003). In fact, application of tDCS for 13 min to the motor cortex can modulate cortical excitability for several hours (Nitsche and Paulus, 2000, 2001). Two cortical areas have been explored in pain studies using tDCS: primary motor cortex and dorsolateral prefrontal cortex (Nitsche et al., 2008; DaSilva et al., 2011). In the most common setup for pain research the anode is positioned over the motor cortex (M1) and the cathode over the supra-orbital area (DaSilva et al., 2011). It has been described that the cortical excitability can be changed up to 40% with this method (DaSilva et al., 2011). Regarding the specific area stimulated in M1, studies with non-invasive brain stimulation have shown better results for facial pain with the stimulation of the hand cortical area (medially located) and more significant improvement of hand pain when the cortical area representing the face (more laterally located) is stimulated. One possible explanation would be the direct effect of tDCS/TMS on the thalamus, which could lead to stimulation of the ventroposteromedial nucleus (VPM), responsible for the nociceptive input from the face (Lefaucheur et al., 2004, 2006; Lefaucheur, 2006).

## Case Presentation

### Subject

A 62-year-old woman was recruited by the Headache and Orofacial Pain Effort (H.O.P.E.) laboratory at the University of Michigan to participate in an ongoing study investigating the effects of the tDCS in the μ-opioidergic system. She had a history of herpes zoster in 2008, with severe pain, affecting the distribution of the left ophthalmic (V1) and maxillary (V2) divisions of the trigeminal nerve. The pain persisted after the complete healing of the initial lesions, leading to a diagnosis of post-herpetic neuralgia. During the baseline evaluation, she described the pain as constant, spontaneous, throbbing, aching, heavy, and hot-burning. The average pain intensity was four out of ten and the average of the unpleasantness associated with the spontaneous pain was six out of ten. The pain was alleviated by sleep and massage and aggravated by sleepiness, stress, and alcohol. The patient reported eye dryness and nasal congestion related to her pain. The symptoms could not be triggered with heat, cold, touch, or chewing. Her pain was not associated with nausea, vomiting, photophobia, or headache. The patient rated the levels of social interaction (0 = isolation, 10 = social gathering), attention (0 = inattention, 10 = high awareness), and anxiety (0 = least, 10 = most) at two, three, and six out of ten, respectively, during the spontaneous pain. She had been treated with amitriptyline 10 mg once a day and pregabalin 50 mg twice a day, with only partial control of her pain. The scores of the McGill Pain questionnaire (MPQ) descriptors during the baseline evaluation were: 24 (sensory), 5 (affective), 2 (evaluative), and 7 (miscellaneous). The pain rating index (PRI) was 38 and the present pain intensity (PPI) was three (distressing). All procedures reported were carried out in accordance with the bioethical rules for studies involving human beings of the WMA (World Medical Association, 2012) – Declaration of Helsinki (2008). The protocol of this study was previously approved by the University of Michigan Investigational Review Board for Human Subject Use and by the Radioactive Drug Research Committee of the US Food and Drug Administration. The patient gave written informed consent prior to the participation in the study.

### Neuroimaging

We used a radiotracer with specific affinity for μORs, [^11^C] carfentanil. The participant underwent one baseline and one tDCS90-min PET scan using a Siemens (Knoxville, TN, USA) HR + scanner in 3D mode (reconstructed images have a full-width at half maximum (FWHM) resolution of approximately 5.5 mm-in-plane and 5.0 mm axially). Synthesis of high specific activity [^11^C]carfentanil (>2000 Ci/mmol) was produced by the reaction of [^11^C]methyliodide and a non-methyl precursor (Dannals et al., 1985; Jewett, 2001). Each [^11^C]carfentanil dose (10–15 mCi, ≤0.03 μg/kg) was administered at 50% as a bolus with the remnants constantly injected across the session to reach normalized tracer levels approximately 35 min after tracer administration.

Positron emission tomography images were reconstructed using interactive algorithms into a 128 × 128 pixel-matrix in a 28.8 cm diameter field of view (FOV). Twenty-eight image frames were obtained and co-registered to one another. They were corrected for motion and decay (Minoshima et al., 1993). Dynamic image data for each scan were converted on a voxel-by-voxel basis into two sets of parametric images: First, a tracer transport measure (K1 ratio) used for co-registration and normalization procedures; and second, a receptor-related measure, distribution volume ratio (DVR, equal to *B*_max_/*K*_d_ + 1 or binding potential at equilibrium (BP_ND_) + 1). These two measures were estimated using a modified Logan graphical analysis using the occipital cortex as the reference region (Logan et al., 1996).

A T1-weighted anatomical MRI scan was acquired on a 3 T scanner (General Electric, Milwaukee, WI, USA). The MRI acquisition utilized the following sequence parameters: axial spoiled-gradient recalled (SPGR) 3D acquisition, 15.63 bandwidth, repetition time [TR] = 9.2 ms, echo time [TE] = 1.9 ms, inversion recovery preparation 500 ms, flip angle = 15°, 25/26 FOV, number of excitations [NEX] = 1, 144 contiguous slices, 1.0 mm slice thickness, 256 × 256 matrix.

Images were anatomically standardized into template space using Statistical Parametric Mapping (SPM8) software by (A) co-registering the MR scan and K1 scans; (B) normalizing the MR scan to the Montreal Neurologic Institute (MNI) template brain using DARTEL; and (C) applying the resulting deformation matrix to the PET images. Co-registration and normalization accuracy was verified by comparing the transformed MR and PET images to the MNI atlas template.

### Transcranial direct current stimulation

Both placebo and active tDCS were applied during the second PET scan. The placebo tDCS was applied during the early phase of the exam (15–35 min), while the active tDCS during the late phase (60–80 min). This sequence was adopted to avoid carry-over effects from the placebo tDCS. In active stimulation 2 mA of tDCS was applied for 20 min. The anode was placed over the area corresponding to the primary motor cortex (M1) while the cathode was positioned over the supra-orbital region. For placebo tDCS, the same method was used; however current was applied only for 30 s. This has been demonstrated to be a reliable method of sham stimulation (Gandiga et al., 2006) as sensations arising from tDCS treatment are observed usually at the beginning of application. The impedance was controlled under 5 kΩ during the whole period of active stimulation to avoid abnormal increase of the overall resistance and consequently heat that could potentially burn the patient. The tDCS protocol used in this study is fully explained in a stepwise manner by our scientific team in DaSilva et al. (2011). Due to the space restrictions, considering the stimulation inside the PET scanner, a special system was developed to add more solution to the sponges when needed. This system consisted of two syringes, each one connected to one sponge by two small tubes. Each electrode was positioned inside a 35 cm^2^ sponge, that was soaked with approximately 12 mL of saline solution (6 mL per side) before the PET and up to 12 mL during the procedure. We used saline solutions with lower concentrations of NaCl (15 mM) (DaSilva et al., 2011).

### Quantitative sensory testing

In this study we controlled the effects of tDCS on the thermal perception as assessed by the Quantitative Sensory Testing (QST) in three moments during the second PET: before starting the scan, in the period between sham and active tDCS (approximately 40–60 min) and after the scan. For this purpose, a QST protocol, consisting of thermal pain thresholds for cold and hot stimuli, was performed using a Thermal Sensory Analyzer TSA 2001-II (Medoc, Israel) (Yarnitsky and Sprecher, 1994; Bachmann et al., 2010). The thermal stimuli were applied upon V3, bilaterally, and dorsal radial area of both hands. Each stimulus was applied for three consecutive times and the average was calculated.

## Results

**Figure 1:**
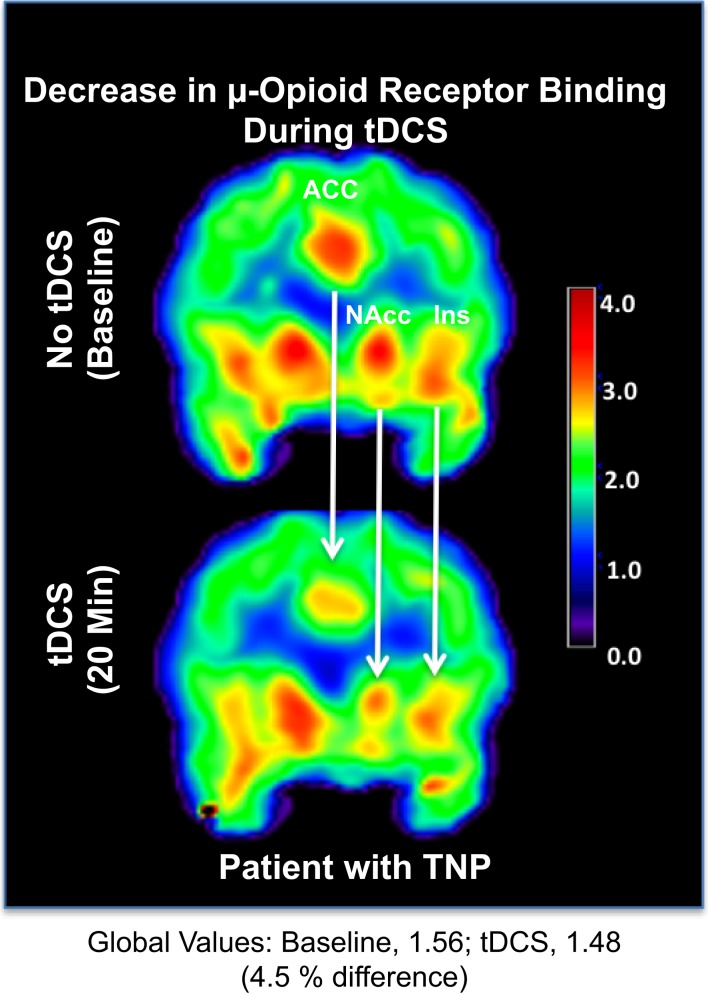
**Decrease in μ-opioid receptor binding associated with transcranial direct current stimulation**. Upper panel: μOR BP_ND_ during the baseline PET. Lower panel: μOR BP_ND_ during active tDCS. ACC, anterior cingulate cortex; NAc, nucleus accumbens; Ins, insula.

**Figure 2:**
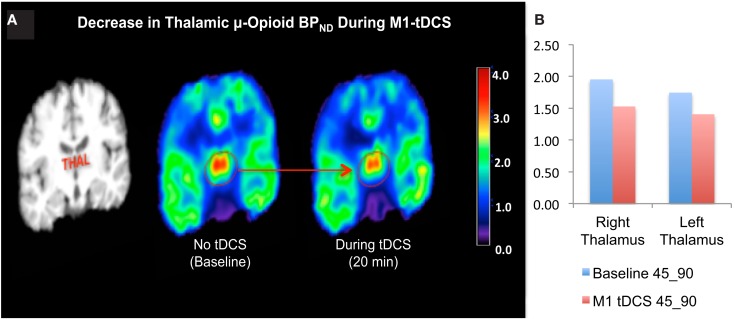
**(A)** Decreased thalamic μ-opioid receptor availability during active tDCS, represented in the coronal plane. **(B)** Bar chart illustrating the μ-opioid receptor binding potential in the right and left thalamus during the late phase of the first and second PET scans.

Levels of μOR BP_ND_ in our trigeminal neuropathic pain patient (TNP) patient during a single tDCS application immediately induced significant decrease in μOR binding in many (sub)cortical pain-matrix structures, including nucleus accumbens (NAc), anterior cingulate cortex (ACC), insula (Ins), and thalamus (Thal; Figures 1 and 2). For instance, the M1-tDCS montage considerably decreased μOR binding in the posterior thalamus (R: 21.5%; L: 19.54%), compared to sham tDCS (R: 2.2%; L: 4.7%).

No significant changes were observed in the clinical pain levels related to tDCS. The pain as assessed by the visual analog scale (VAS) was four out of ten before the second PET, three after placebo tDCS, and returned to four after the PET. Regarding the QST, a significant increase in the temperature for heat threshold was observed in the left V3 after sham tDCS and when comparing baseline and active tDCS. On the hand, the temperature for cold threshold showed a significant decrease in the left V3 after active tDCS but not after placebo tDCS. When comparing the cold threshold after active tDCS to the baseline threshold (before starting the PET scan), there was a reduction of the temperature at which cold pain was detected in the left V3 of approximately 36.2%. Significant changes in the heat and cold thresholds associated with sham and active tDCS were also observed in other regions, such as right V3, and right hand. The QST results are presented in the Table 1.

**Table 1:** **Variations in the heat and cold thresholds related to placebo and active tDCS**.

Region	Heat/cold	Before PET		After placebo	tDCS	After active tDCS		Significance level (*p*)		
		Mean	SD	Mean	SD	Mean	SD	Baseline × placebo tDCS	Baseline × active tDCS	Placebo × active tDCS
Left V3	Heat	35.8	0.4	42.7	1.9	44.9	0.9	***p* < 0.001**	***p* < 0.001**	*p* > 0.05
Right V3	Heat	42.2	0.8	43.6	1.1	44.1	1.1	NS	NS	NS
Left hand	Heat	46.3	1.0	46.3	1.0	43.5	2.1	NS	NS	NS
Right hand	Heat	44.2	1.0	40.0	1.1	44.2	1.9	***p* < 0.05**	*p* > 0.05	*p* > 0.05
Left V3	Cold	23.7	2.3	22.7	2.5	15.1	3.2	*p* > 0.05	***p* < 0.05**	***p* < 0.05**
Right V3	Cold	20.1	3.8	20.2	3.8	9.5	5.9	*p* > 0.05	***p* < 0.05**	***p* < 0.05**
Left hand	Cold	15.1	6.8	18.3	6.8	12.9	4.3	NS	NS	NS
Right hand	Cold	14.1	3.4	12.6	2.3	18.0	9.1	NS	NS	NS

*Statistical significance (in bold) was defined at *p* < 0.05 (one-way ANOVA followed by Tukey’s test)*.

## Discussion

To our knowledge, this is the first study showing an immediate reduction in theμOR binding in response to an acute motor cortex neuromodulation, suggesting that the analgesic effect of M1-tDCS is possibly due to direct increase of endogenous opioid release.

Endogenous opioid systems have long been implicated in regulating pain nociceptive signals, with μORs being the primary mediators of opiate analgesia, but also the rewarding and tolerance-producing effects of opiates (Sora et al., 1997). Both elements, endogenous opioid release and μOR concentrations, are therefore critical elements for the understanding of chronification and alleviation of pain in TNP patients. The first direct evidence of regional endogenous μ-opioid activation during sustained experimental trigeminal pain in healthy humans was published by Zubieta et al. (2001) using PET, measured with external imaging as reductions in the *in vivo* availability of μORs BP_ND_ quantified with [^11^C]carfentanil. Acute reductions in μOR BP_ND_ were observed in the PAG, thalamus, hypothalamus, NAc, ventral pallidum, amygdala, insula, and dorsal anterior cingulate (dACC), correlating with suppression of sensory and affective qualities of the pain challenge.

The investigation of the response of the endogenous opioid system to TNP and its neuromodulation models is of importance to understand the mechanisms in place to regulate the pain experience. This information is key to better predict the varied responses of TNP patients to therapeutic interventions. Jones et al. (1994, 1999) utilized [^11^C]diprenorphine, a non-selective opioid radiotracer, to examine the *in vivo* availability of opioid receptors in a small group of patients diagnosed with rheumatoid arthritis and trigeminal neuralgia before and 3 weeks to 3 months after treatment and pain relief. Substantial reductions in cortical and subcortical opioid receptor availability were observed prior to treatment at resting state (baseline), which were reversed after pain relief. Similar results were obtained with [^11^C]diprenorphine in four central post stroke pain patients and in a patient with a pontine infarction and pain (Willoch et al., 1999, 2004), suggesting a dysregulation of central opioid mechanisms at baseline in response to chronic pain, regardless of pain etiology. Interestingly, in a study with eight refractory neuropathic pain patients, postoperative (invasive) motor cortex stimulation induced decreases of [^11^C]diprenorphine binding in the anterior mid-cingulate cortex (MCC) and PAG, which were significantly correlated with pain relief (Maarrawi et al., 2007b). The authors suggested that the decrease in binding of the exogenous ligand was possibly due to receptor occupancy by enhanced release of endogenous opioids. This analgesic mechanism is highly associated with M1 cortex stimulation, at least with rTMS, since it is blocked with naloxone injection (Taylor et al., 2012). tDCS over M1 induces immediate changes in thermal sensory percepts in health subjects, especially cold (Bachmann et al., 2010). In addition, it produces long lasting pain relief in chronic pain patients, including TNP (Lima and Fregni, 2008). Recently, it was reported that acute tDCS modulates functional connectivity depending on its polarity (Polania et al., 2011). Anodal stimulation over M1 with contralateral frontocortical cathode placement (our protocol) immediately increases functional coupling between ipsilateral M1 and thalamus. On the contrary, cathodal tDCS over M1 decreases functional coupling between ipsilateral M1 and contralateral putamen.

The findings above hint why the anode M1/cathode orbitofrontal electrode montage results in optimal modulation of pain-matrix hyperactivity, specially the thalamus, which underlies chronic pain. Here, in our case report data with TNP, the same active M1-tDCS montage considerably decreased μOR binding in the posterior thalamus (Figure 2). Nonetheless, it is possible that an additional opioid release might have been prevented by a potential carry-over effect related to the sham stimulation.

Remarkably, the single tDCS application immediately improved 36.2% the threshold for experimental cold pain in the allodynic V3 area (baseline: 23.7°C ± 2.3; placebo tDCS: 22.7°C ± 2.5; active tDCS 15.1°C ± 3.2), but not the TNP patient’s clinical pain (baseline: 4, VAS 0-10); placebo tDCS: 3; active tDCS: 4). Suggesting that the immediate opioidergic effects of a single tDCS session are subclinical, and repetitive sessions are necessary to revert ingrained neuroplastic changes related to the chronic TNP suffering (see next paragraph). This is in agreement with the results from multiple clinical tDCS studies, showing a direct relationship of patients’ clinical pain improvement with the number of tDCS sessions (Lima and Fregni, 2008).

## Concluding Remarks

This case report represents a change of paradigm, as we directly modulated the same opioid mechanisms under study by applying novel neuroimaging and neuromodulatory tools. Future studies are necessary to confirm our results, and to investigate further the effects of tDCS on the endogenous opioid system in a larger cohort of patients.

## Conflict of Interest Statement

Conflict of Interest Statement: The authors declare that the research was conducted in the absence of any commercial or financial relationships that could be construed as a potential conflict of interest.
